# Acute Blood Pressure-Lowering Effects of Nitrogen Dioxide Exposure From Domestic Gas Cooking Via Elevation of Plasma Nitrite Concentration in Healthy Individuals

**DOI:** 10.1161/CIRCRESAHA.120.316748

**Published:** 2020-06-16

**Authors:** Christopher N. Floyd, Fawzia Shahed, Frances Ukah, Karen McNeill, Kevin O’Gallagher, Charlotte E. Mills, Dimitris Evangelopoulos, Shanon Lim, Ian Mudway, Benjamin Barratt, Heather Walton, Andrew J. Webb

**Affiliations:** 1 From King’s College London (KCL) British Heart Foundation (BHF) Centre, School of Cardiovascular Medicine and Sciences, Department of Clinical Pharmacology, National Institute for Health Research (NIHR) Biomedical Research Centre (BRC), Clinical Research Facility (CRF), Guy’s and St Thomas’ NHS Foundation Trust (GSTFT), London, UK (C.N.F., F.S., F.U., K.M., K.O.G., A.J.W.); 2 Nutritional Sciences, School of Life Course Sciences, King’s College London, UK, Food and Nutritional Sciences, School of Chemistry, Food and Pharmacy, University of Reading, UK (C.E.M.); 3 NIHR Health Protection Research Unit (HPRU) on Health Impacts of Environmental Hazards at KCL in Partnership with Public Health England (PHE) and Imperial College London (ICL), UK (D.E., S.L., I.M., B.B., H.W.).

**Keywords:** air pollution, blood pressure, nitrite, nitrogen dioxide, particulate matter

**Meet the First Author, see p 706**

Air pollution is a major cause of cardiovascular and all-cause mortality. Disentangling the relative contributions of pollutants is challenging, as epidemiological data measuring exposure to one (eg, nitrogen dioxide [NO_2_]) is inevitably confounded by exposure to others (eg, particulate matter). Animal studies suggest that inhaled NO_2_ has the potential to increase plasma [nitrite]^1^; a chemical originally considered to be physiologically inert before we found that its reduction to nitric oxide protects the myocardium against ischemia-reperfusion injury and lowers blood pressure in humans.^2^

We conducted an acute, randomized, controlled, crossover study to assess the impact of 90 minutes exposure to NO_2_ (from sitting next to a domestic gas cooker with gas hobs lit and uncovered) versus control (room air) on plasma [nitrite] (primary end point) and blood pressure (secondary end points) in 12 healthy participants. All underwent both interventions/visits (interval 7–108 days) in a computer-generated randomized order. Baseline characteristics (mean±SD): 26±4years, 10/12 female, body mass index 21.9±3.0 kg/m^2^, systolic blood pressure 113.8±7.9 mm Hg, diastolic blood pressure 72.8±5.7 mm Hg. The exposure phase was followed by a 90 minutes washout phase at background [NO_2_]. Participants fasted for 12 hours before each visit and received 250 mL low-nitrate water at time 0 h/1.5 h. The study was powered for a difference in plasma [nitrite] of 27±40 nmol/L on repeated-measures, 2-way ANOVA (α, 0.05 and β, 0.2) following D’Agostino-Pearson normality-confirmation, with Sidak post-test (GraphPad Prism v8.2.1).^3^

The data that support the findings of this study are available from the corresponding author upon reasonable request.

Relative to control, exposure increased ambient [NO_2_]: 276.3±38.5 versus 27.6±2.8 ppb (*P*<0.001). Plasma [nitrite] was increased through both the 90 minutes NO_2_ exposure and 90 minutes washout (*P*<0.001; Figure [A]). NO_2_ exposure decreased both systolic blood pressure and diastolic blood pressure (both *P*<0.001; Figure [B] and [C]). The largest decrease in systolic blood pressure relative to control occurred at 45 minutes (4.6 mm Hg [95% CI, 0.2–8.9]; *P*=0.032) and 90 minutes (5.5 mm Hg [95% CI, 1.2–9.9]; *P*=0.005). The effect of NO_2_ on diastolic blood pressure was maximal at 45 minutes (5.7 mm Hg [95% CI, 0.9–10.5]; *P*=0.009).

**Figure.**
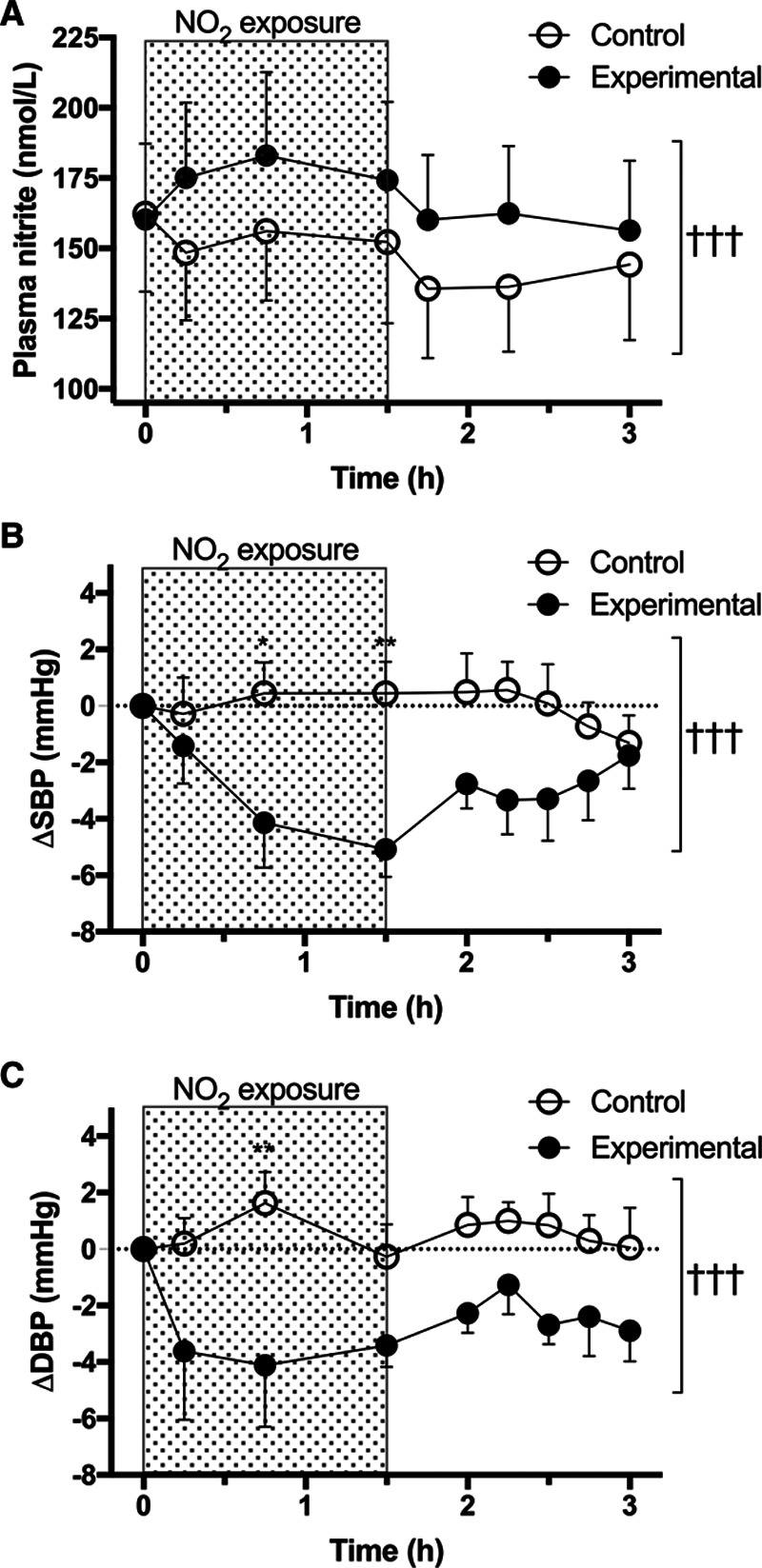
**Effect of nitrogen dioxide (NO_2_) exposure on plasma [nitrite] and blood pressure.** Plasma [nitrite] (**A**), systolic blood pressure (**Δ**SBP; **B**), diastolic blood pressure (**Δ**DBP; **C**). Comparison between experimental and control shown as †††*P*<0.001 and individual timepoints as *P<0.05 and **P<0.01. Data expressed as mean±SEM (n=12).

The temporal relationship between the increase in plasma [nitrite] and systolic blood pressure/diastolic blood pressure reduction (≈5 mm Hg) is consistent with studies investigating dietary nitrate.^2^ Furthermore, whilst the level of NO_2_ exposure (276.3±38.5 ppb) was ≈2.5-fold greater than recommended limits for exposure (eg, World Health Organization guideline 105 ppb 1-hour mean), it is less than that recorded adjacent to busy roads or in some domestic kitchens (≈2000 ppb). Our model is, therefore, conservative relative to real-world exposure.

Previously, an increase in plasma [nitrite] at 2 hours following diesel exhaust inhalation was thought to be due to particulate matter-mediated induction of inflammatory pathways.^3^

However, our data suggest a more rapid increase in plasma [nitrite] which favors chemical conversion from NO_2_ (eg, via a nitrous acid intermediary) and presents a plausible mechanism through which inhaled NO_2_ increases plasma [nitrite].^1^ This novel ecophysiological NOx cycle may directly feed into the established nitrate-nitrite-nitric oxide pathway and contribute nitric oxide-mediated cardiovascular effects.^2^ Adverse respiratory effects of inhaled NO_2_ were not investigated here.^1^

These data must be considered in the context of the strong epidemiological association between NO_2_ exposure and cardiovascular mortality.^1^ However, particulate matter-free NO_2_ does not appear to impair either vascular function, fibrinolysis, or affect heart rate variability in patients with coronary heart disease: parameters adversely affected by increased ambient NO_2_ exposure in epidemiological studies.^4,5^ This study expands our understanding of how inhaled NO_2_ might impact the cardiovascular system, and the role of diet in disease: it is not just what you eat, but how you cook it that matters.

## Sources of Funding

King’s Together Multi and Interdisciplinary Research Scheme (Wellcome Trust Institutional Strategic Support Fund [204823/Z/16/Z]). H. Walton and D. Evangelopoulos’s posts part-funded by NIHR-HPRU on Health Impacts of Environmental Hazards at KCL in Partnership with PHE and ICL, London, UK. Internal infrastructure financial support: KCL-BHF Centre, NIHR-CRF/NIHR-BRC at GSTFT/KCL. Views expressed are authors’ (not necessarily NHS/NIHR/DHSC/PHE).

## Disclosures

None.
